# JNK modifies neuronal metabolism to promote proteostasis and longevity

**DOI:** 10.1111/acel.12849

**Published:** 2019-02-27

**Authors:** Lifen Wang, Sonnet S. Davis, Martin Borch Jensen, Imilce A. Rodriguez‐Fernandez, Cagsar Apaydin, Gabor Juhasz, Bradford W. Gibson, Birgit Schilling, Arvind Ramanathan, Sina Ghaemmaghami, Heinrich Jasper

**Affiliations:** ^1^ The Buck Institute for Research on Aging Novato California; ^2^ Genentech Inc. South San Francisco California; ^3^ Department of Anatomy, Cell and Developmental Biology Eotvos Lorand University Budapest Hungary; ^4^ Institute of Genetics Biological Research Center of the Hungarian Academy of Sciences Szeged Hungary; ^5^ Department of Biology University of Rochester Rochester New York

**Keywords:** aging, Jun‐N‐terminal kinase, lifespan, metabolism, protein turnover, proteostasis

## Abstract

Aging is associated with a progressive loss of tissue and metabolic homeostasis. This loss can be delayed by single‐gene perturbations, increasing lifespan. How such perturbations affect metabolic and proteostatic networks to extend lifespan remains unclear. Here, we address this question by comprehensively characterizing age‐related changes in protein turnover rates in the *Drosophila* brain, as well as changes in the neuronal metabolome, transcriptome, and carbon flux in long‐lived animals with elevated Jun‐N‐terminal Kinase signaling. We find that these animals exhibit a delayed age‐related decline in protein turnover rates, as well as decreased steady‐state neuronal glucose‐6‐phosphate levels and elevated carbon flux into the pentose phosphate pathway due to the induction of glucose‐6‐phosphate dehydrogenase (G6PD). Over‐expressing G6PD in neurons is sufficient to phenocopy these metabolic and proteostatic changes, as well as extend lifespan. Our study identifies a link between metabolic changes and improved proteostasis in neurons that contributes to the lifespan extension in long‐lived mutants.

## 1 INTRODUCTION

Studies in model organisms have generated significant insight into the genetic factors and environmental conditions that influence, cause, and prevent aging (Kennedy et al., 2014; Lopez‐Otin, Blasco, Partridge, Serrano, & Kroemer, 2013). Stress‐induced damage to biological macromolecules, altered energy metabolism, deregulation of processes ensuring cellular and tissue integrity, and loss of coordination of systemic physiological control mechanisms are among the drivers of the general loss of homeostasis observed in aging organisms (Riera & Dillin, 2015; Taylor & Dillin, 2011). Strikingly, single‐gene mutations have been identified that affect evolutionarily conserved signaling pathways and result in a coordinated delay of processes that contribute to the age‐related loss of homeostasis, thus significantly extending lifespan (Lopez‐Otin et al., 2013; Partridge, Alic, Bjedov, & Piper, 2011). However, the exact molecular mechanisms that link these mutations to their longevity‐promoting outcomes remain unclear. Characterizing the perturbation of interconnected downstream processes by longevity mutations is thus likely to provide significant insights into the aging process and to allow identifying novel longevity‐promoting interventions.

Changes in cellular metabolism and protein homeostasis (proteostasis) are likely central to the effects of many lifespan‐extending interventions. Mitochondrial activity and metabolic status are often changed by such interventions, reducing the production of reactive oxygen species (ROS) and other damaging metabolic by‐products (Avanesov et al., 2014; Houtkooper, Williams, & Auwerx, 2010; Riera & Dillin, 2015). A breakdown in proteostasis, in turn, is a central part of the normal aging process and is also evident in age‐related protein aggregation diseases of the brain, such as Alzheimer's disease (AD) (Labbadia & Morimoto, 2015; Taylor & Dillin, 2011). Improving proteostasis in model organisms, by increasing the expression of molecular chaperones, or by treating with compounds that reduce protein aggregates, can extend lifespan significantly (Alavez, Vantipalli, Zucker, Klang, & Lithgow, 2011; Taylor & Dillin, 2011; Tower, 2011). Similarly, increasing expression or function of the protein degradation machinery can significantly increase lifespan (Chondrogianni, Georgila, Kourtis, Tavernarakis, & Gonos, 2015; Juhasz, Erdi, Sass, & Neufeld, 2007; Vilchez et al., 2012), while reducing the rate of protein synthesis through depletion of translation initiation factors, ribosomal proteins, or ribosomal protein regulators increases lifespan in yeast, worms, and flies (Hansen et al., 2007; Steffen et al., 2008; Syntichaki, Troulinaki, & Tavernarakis, 2007; Wang, Cui, Jiang, & Xie, 2014). Lifespan can further be extended in all tested model organisms by inhibiting insulin‐IGF signaling (IIS) and Tor, a central nutrient sensor that influences protein homeostasis by a number of mechanisms (Kenyon, 2005; Lopez‐Otin et al., 2013; Partridge et al., 2011). Together, these studies point to metabolic perturbations and the loss of proteostasis as central drivers of deleterious aging phenotypes and age‐related diseases (Labbadia & Morimoto, 2015; Lopez‐Otin et al., 2013; Taylor & Dillin, 2011).

One of the most prominent proteostatic disruptions that are observed in aging cells and tissues is a global decrease in protein turnover rates. Age‐related increases in protein half‐lives have been documented in a number of species, including nematodes (Prasanna & Lane, 1979), rats (Lewis, Goldspink, Phillips, Merry, & Holehan, 1985), and humans (Young, Steffee, Pencharz, Winterer, & Scrimshaw, 1975) and may drive aberrant protein accumulation and post‐translational modification patterns (Rattan, 2010). These alterations can lead to a variety of irreversible protein damage, rendering cells dysfunctional and potentially accelerating the aging process. Reversing this age‐induced decline in protein turnover rates could be a potential downstream mechanism by which longevity‐promoting mutations extend lifespan. However, whether and how cellular proteostasis is influenced by such mutations is unclear, and the molecular mechanism(s) of the age‐associated decline in protein turnover are incompletely understood.

**Figure 1:**
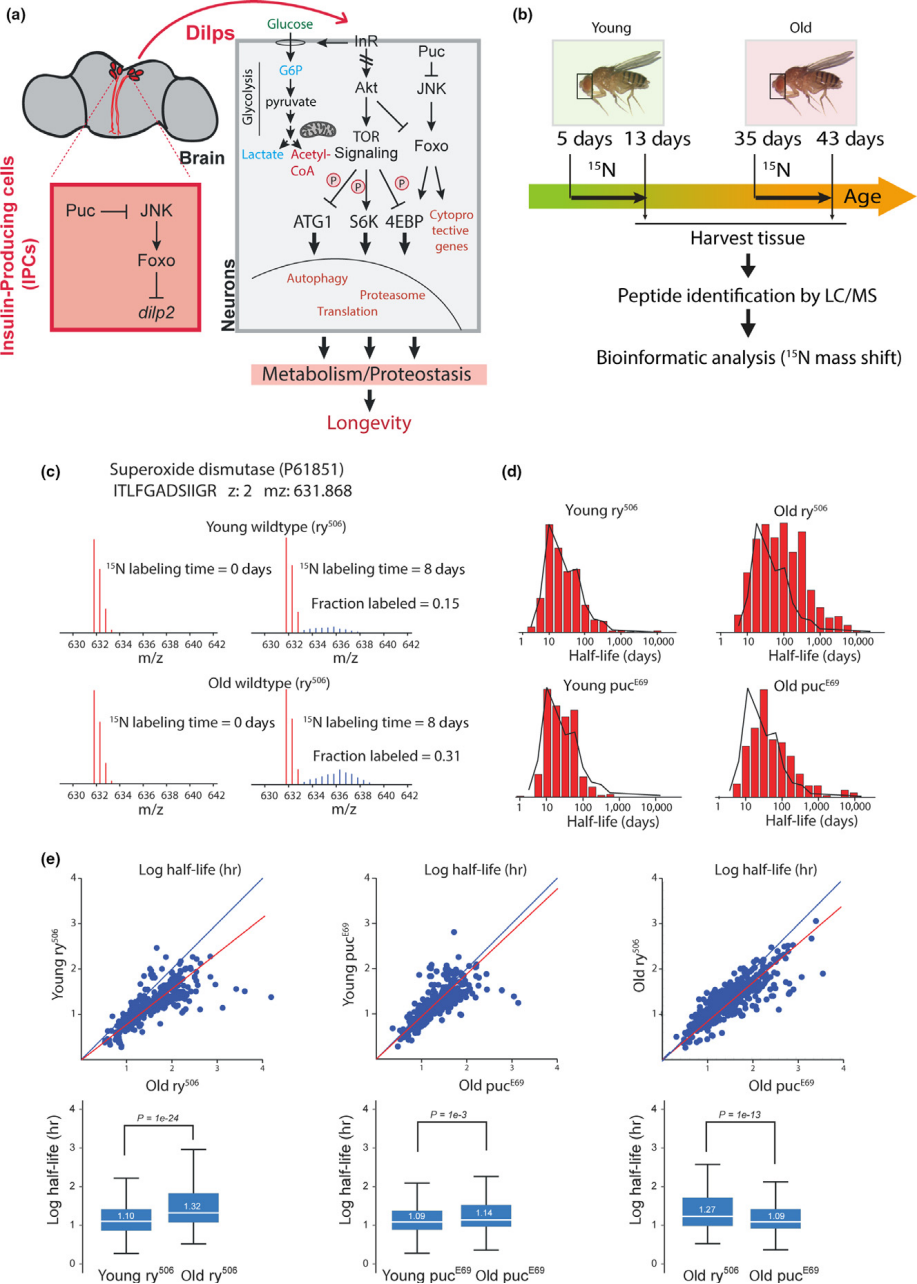
Age‐related changes in protein turnover rates are reduced in heads of *puc* heterozygotes. (a) Model for JNK‐mediated regulation of metabolic homeostasis and lifespan extension. JNK‐mediated repression of insulin‐like peptide 2 (ILP2) expression in insulin‐producing cells (IPCs) of the fly brain results in systemic repression of insulin signaling. This, in turn, promotes FOXO activity and reduces Tor activity, promoting autophagy and limiting translation. JNK‐induced cytoprotective gene expression in the periphery contributes to longevity in JNK gain‐of‐function conditions. How such conditions influence changes in metabolic and protein homeostasis globally remains unclear. (b) Experimental approach to comprehensively characterize protein turnover rates in fly heads. Young (5‐day‐old) and old (35‐day‐old) flies were fed a sugar/yeast diet for 8 days in which ^15^N‐pre‐labeled yeast was the only nitrogen source. Proteins were isolated from whole heads and subjected to mass spectrometry. (c) Example for protein turnover analysis from mass spectrometry data. *m/z* distribution of peptide P61851 derived from superoxide dismutase (SOD) in heads of young and old *ry* flies. Left panels: flies provided with an all ^14^N diet (^14^N yeast); Right panels: flies that were fed ^15^N‐labeled yeast for 8 days. Natural isotopic distribution of the peptide is shown in red. Distribution of ^15^N‐labeled peptide is shown in blue. The fraction labeled was calculated as described in Materials and Methods. (d) Distribution of protein half‐lives from heads of young (5 days) and old (35 days) *ry* or *puc* mutant flies. Note the shift toward longer protein half‐lives in old *ry* heads, but not in old *puc* mutant heads. (e) Comparison of protein half‐lives from heads of young (5 days) and old (35 days) *ry* or *puc* mutant flies. Each dot represents one protein. Note the deviation toward longer half‐lives (away from the blue diagonal with slope 1) in old *ry* heads but not in old *puc* heads. The blue lines indicate the theoretical identity line, and the red line indicates the best linear fit to the data. The box plots indicate the interquartile (box) and the complete (whiskers) range of the data excluding outliers (>2*SD). In the boxplots, the data have been limited to the subset shared between the compared sets. The white line and numbers indicate the median values. Comparisons and *p* value measurements were conducted by the Mann–Whitney *U* test

It further remains to be established how other metabolic, stress, and repair responses induced by longevity mutations are integrated with proteostatic changes to influence lifespan. Flies with increased Jun‐N‐terminal kinase (JNK) activity, for example, show a robust extension of lifespan that is mediated by the inhibition of insulin‐like peptide expression in insulin‐producing cells (Wang, Bohmann, & Jasper, 2003, 2005 ), by the induction of insulin resistance in peripheral tissues, as well as by JNK‐mediated improvement of cell‐autonomous protection and repair capabilities (Biteau, Karpac, Hwangbo, & Jasper, 2011). While increased JNK signaling activity thus induces metabolic changes and cytoprotective gene expression that are representative of IIS inhibition (Figure 1a), these changes may also be accompanied by the induction of proteostatic changes (Wu, Wang, & Bohmann, 2009; Xu, Das, Reilly, & Davis, 2011). How these different aspects of the response to JNK are integrated to achieve longevity remains unclear.

Addressing these questions requires new approaches that comprehensively characterize the impact of longevity‐promoting interventions on metabolism and proteostasis in a complex organism. Highly sensitive and high‐throughput mass spectrometry technologies are beginning to allow such comprehensive assessments of protein turnover and metabolic flux and can be complemented by high‐throughput characterization of cellular transcriptomes. Here, we employ such technologies to characterize the metabolic and proteostatic effects of longevity‐promoting JNK gain‐of‐function conditions on *Drosophila* brains. Our results confirm that in wild‐type animals, protein turnover rates are globally reduced as a function of age, and we find that this is mitigated in long‐lived mutants for the JNK‐specific phosphatase Puckered (*puc^E69^*). Using untargeted metabolite profiling by LC‐MS/MS, flux analysis based on the ^13^C‐glucose uptake, and transcriptome profiling, we further find that JNK triggers a shift in neuronal carbon flux toward the pentose–phosphate pathway (PPP) by inducing the expression of glucose‐6‐phosphate dehydrogenase (G6PD), increasing NADPH production and reducing oxidative stress. Our data suggest that this metabolic reprogramming is responsible for the proteostatic and longevity effects of the *puc^E69^* mutation, as these phenotypes can be mimicked by increasing expression of G6PD in neurons. Our combined proteomic and metabolomic analysis suggests that JNK activation extends lifespan through metabolic reprogramming that promotes neuronal proteostasis.

## 2 RESULTS

### 2.1 Analysis of proteome dynamics in long‐lived flies

To characterize changes in proteostasis and cellular metabolism that are associated with longevity, we sought to analyze age‐related changes in protein turnover and steady‐state metabolite concentrations as well as metabolic flux in wild‐type and long‐lived flies. We chose to focus on the brain as a highly metabolic tissue in which the age‐related decline in proteostasis manifests itself in a range of age‐related diseases. In flies, rapidly isolating frozen heads in bulk is feasible, providing an opportunity to collect relatively homogeneous tissue samples in large enough amounts for proteomic and metabolomic analysis.

As long‐lived mutants, we chose animals carrying a loss‐of‐function allele for *puckered* (*puc^E69^*). Puckered encodes a JNK phosphatase and *puc^E69^* heterozygotes are long‐lived as a consequence of elevated JNK activity (Figure 1a; Biteau et al., 2011; Libert, Chao, Zwiener, & Pletcher, 2008; Wang, Bohmann, & Jasper, 2003; Wang, Bohmann, & Jasper, 2005)). We confirmed longevity of the animals we used in a variety of dietary conditions (Supporting Information Figure S1). Independent of the concentration of yeast (as only protein source) in the food, *puc^E69^* heterozygotes lived substantially and significantly longer than isogenic controls (Supporting Information Figure S1; the *ry^506^* mutation is used as a background mutation to track the ry^+P^ element inserted into the *puc* locus; the lifespan analysis and further studies were performed in *puc^E69^* and wild‐type control (OreR) animals crossed out into the *ry^506^* background, rendering both experimental cohorts heterozygous for *ry*).

**Figure 2:**
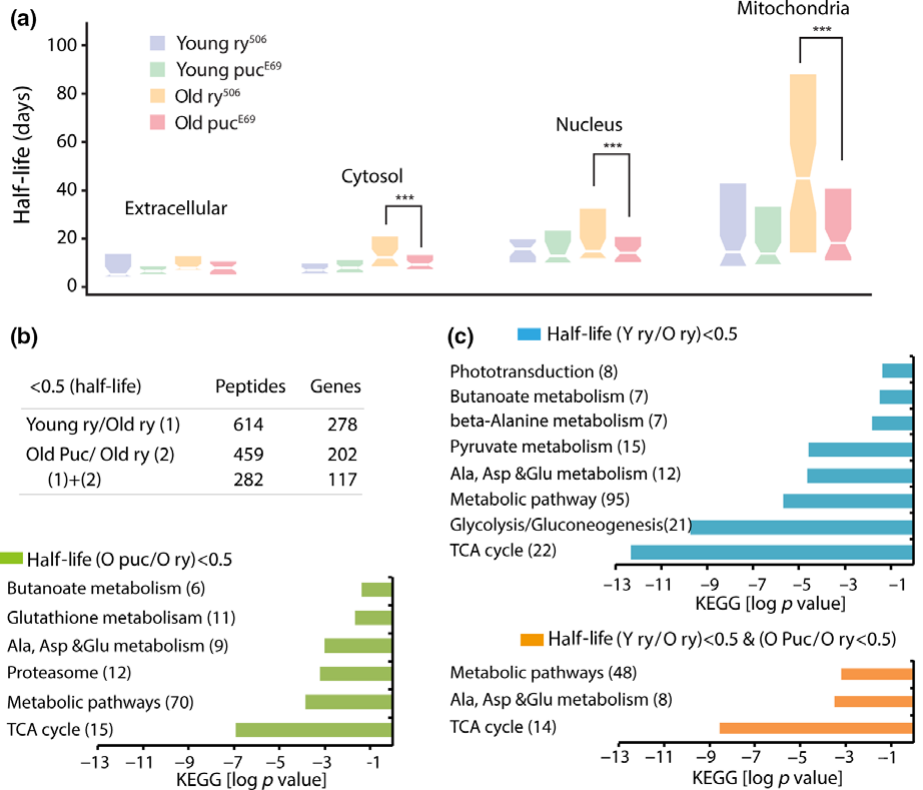
Cellular and functional distribution of proteins with differential half‐lives. (a) Distribution of protein half‐lives in distinct subcellular compartments. Half‐lives of mitochondrial proteins significantly increase in heads of old *ry*, but not in heads of old *puc* flies. *** indicates a *p* value of less than 1e‐4 determined by the Mann–Whitney *U* test. (b, c) Numbers and GO enrichment of identified peptides and corresponding genes with half‐lives that increase more than twofold in heads of old *ry* flies compared old *puc *(b), to young *ry*, or to both (c)

To globally measure protein turnover rates, we performed labeling experiments where near‐uniform ^15^N‐labeled yeast was used as the only nitrogen source for flies. Wild‐type (*ry^506^/+*) controls and *puc^E69 ^*heterozygotes (*puc^E69(ry+)^*, *ry^506^/ry^506^*) were fed a diet in which the only nitrogen source was ^15^N‐labeled yeast for 8 days starting at young (5 days) or old (35 days) ages (Figure 1b,c). Proteins harvested from isolated heads were subjected to tryptic digestion, and isotope incorporation rates were determined using mass spectrometry (Figure 1c, Supporting Information Figure S2). Protein turnover rates were calculated based on averaged relative isotopic abundance for each peptide, using single exponential fitting for the ratio of ^15^N/^14^N distribution (Price, Guan, Burlingame, Prusiner, & Ghaemmaghami, 2010). Using this analytical method for each peptide identified by MS, protein turnover rates of 1,080 protein groups were quantified with high statistical confidence (Figure 1d,e; Supporting Information Tables S1 and S2). In wild‐type flies, protein turnover rates were globally reduced in old heads (comparing 35‐day‐old vs. 5‐day‐old flies; Figure 1e). Reductions in turnover rates were particularly pronounced for long‐lived proteins with half‐lives exceeding 100 days and for proteins residing in the mitochondria (Figures 1e and 2a). Remarkably, this age‐induced reduction in turnover rates was not observed in heads of old long‐lived *puc^E69^* heterozygotes (Figures 1e and 2a).

Consistent with the overrepresentation of mitochondrial proteins, functional classification (gene ontology analysis using Flymine.org) of proteins in which half‐life increases more than twofold between young and old wild‐type animals suggested a significant enrichment of proteins involved in glycolysis and the TCA cycle, as well as other metabolic pathways (Figure 2). Most of these proteins have longer half‐lives in old wild‐type animals compared to old *puc^E69^* mutants, suggesting that an age‐related breakdown in proteostasis particularly affects enzymes involved in critical metabolic pathways and that this decline correlates with shorter lifespan (Figure 2b,c).

### 2.2 Metabolite analysis shows strongly reduced levels of G‐6‐P in puc mutant brains

**Figure 3:**
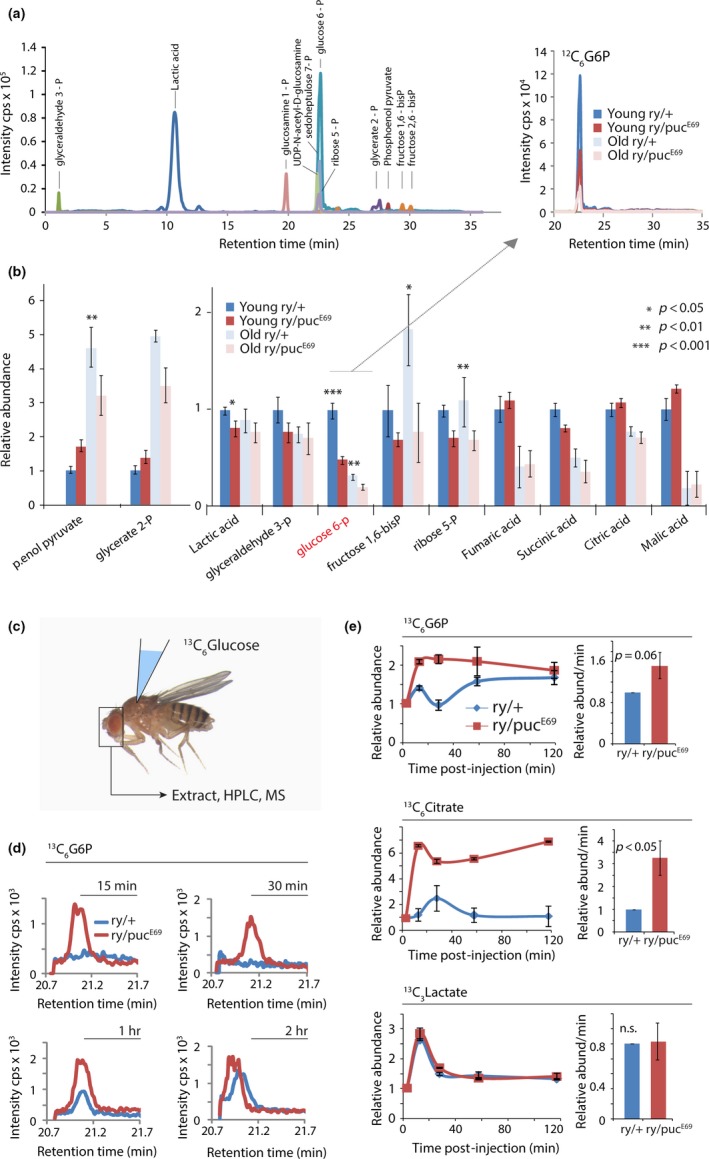
Characterization of steady‐state metabolites and dynamic metabolic flux using ^13^C isotopic tracers in flies. (a) Identification of glycolytic/pentose phosphate pathway (PPP) intermediates in heads of long‐lived *puc* mutants using high‐resolution LC/MS‐based metabolomics. (b) Comparative analysis of glycolytic/TCA//PPP intermediates in steady state. Note lower glucose‐6‐phosphate (G6P) levels in brains of young *puc* mutants compared to *ry^506^*. (c) Injections of ^13^C‐labeled glucose into hemolymph. Fly heads were collected for measurement of the metabolic products of ^13^C‐glucose at 0 min, 15 min, 30 min, 1 hr, and 2 hr after injection. (d) Comparison of ^13^C_6_ G6P levels in brains of young *puc* mutants and *ry^506 ^*at 15 min‐2 hr after injection. ^13^C G6P is detected earlier in *puc *mutants than in *ry* animals. (e) ^13^C_6‐ _G6P, ^13^C_6‐ _citrate and ^13^C_3‐ _lactate show distinct time‐courses over a 2‐hr time period in young long‐lived *puc* mutant and *ry* controls (performed in three biological replicates). Relative rates of metabolic flux (changes in abundance/min at maximum signal) were calculated using the generated abundance curves generated from three independent experiments, using three biological replicates per time point. Relative rates of formation are shown as mean ± *SEM*. Student's *t* test

To explore the extent of the metabolic changes in the brain of *puc^E69^* mutants, we performed metabolite profiling using HPLC/mass spectrometry. We assessed steady‐state metabolite concentrations in the heads of young and old, wild‐type and *puc* heterozygous animals (Figure 3a, Supporting Information Figure S3, Table S3). Compared to *ry^506^* heads, *puc* mutant heads contained reduced glycolytic intermediates at young ages, including glucose‐6‐phosphate and Fructose 1,6 bis phosphate, confirming that *puc* mutant heads exhibit broad metabolic changes (Figure 3b). Particularly, intriguing is the significant reduction in glucose‐6‐phosphate, and the lack of changes in downstream glycolytic intermediates such as lactate, and in TCA cycle intermediates. While these differences were interesting, steady‐state metabolomics are unable to provide insight into how metabolism downstream of glucose‐6‐phosphate is remodeled. We hypothesized that although relative levels of downstream metabolites do not change, the flux through these pathways might still be perturbed. To explore this possibility, we developed an LC/MS‐based assay to analyze metabolic flux using ^13^C‐labeled glucose injection into hemolymph (Figure 3c,d). The amount of ^13^C‐labeled glucose‐6‐phosphate (G6P) detected in fly heads correlates with the dose of ^13^C glucose injected into hemolymph, confirming the feasibility of this approach (Supporting Information Figure S4). Per experiment, we used three biological replicates per time point (5, 15, 30, 60, and 120 min) to measure relative labeling of three metabolites (glucose‐6‐phosphate, lactate, and citrate). We chose these three metabolites to analyze the mitochondrial and nonmitochondrial fates of glucose carbon flux. Three independent experiments were performed to measure the rates of maximum labeling of the three metabolites from labeled glucose. Using this approach, we measured the ^13^C flux through the glycolytic pathway in the brain of *puc^E69^* mutants. Interestingly, we found that long‐lived *puc^E69^* mutants had higher flux rates through glycolysis and the TCA cycle: The rate of ^13^C uptake into G6P and Citrate (but not Lactate) was faster in *puc* heterozygotes than in *ry* controls (Figure 3d,e, Supporting Information Table S4).

### 2.3 JNK induces glucose metabolism genes, including G6PD

**Figure 4:**
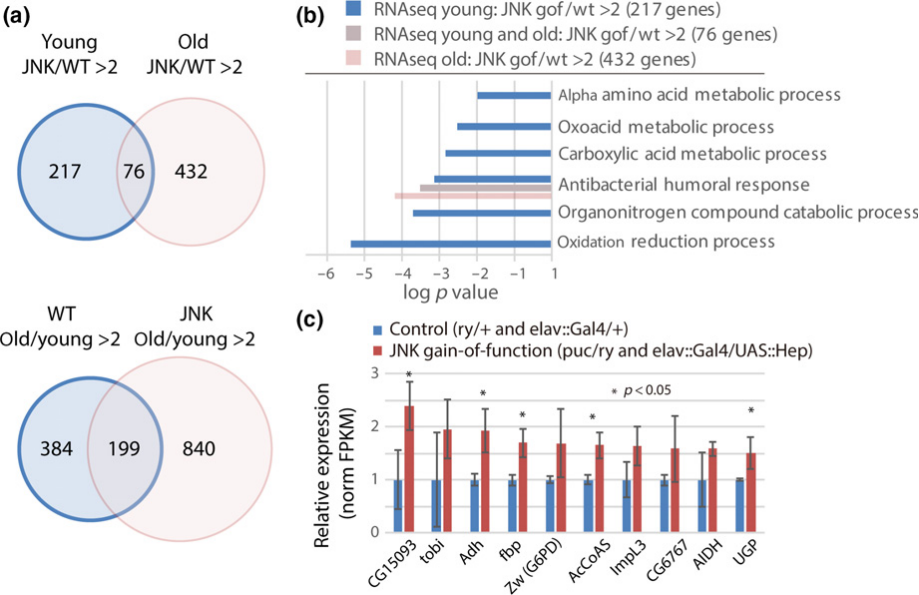
Characterization of JNK‐induced transcriptome changes in brains of young and old flies. (a) Venn diagram summarizing age‐related transcriptional changes in heads of young and old *puc* mutants compared to young or old ry flies, respectively, and of young Hep (JNKK) over‐expressing flies (elav::Ga4/UAS::Hep) compared to controls (elav::Gal4/+). Transcriptomes were obtained by RNAseq. Genes were considered induced when differences in FPKM were at least twofold and FRKM values were at least 10.0. Note that a large subset (199) of genes induced in old wild‐type heads (384) are also induced in *puc* mutants, but that *puc* mutants show a much larger number of transcriptional changes (840 induced genes). (b) GO Analysis of genes induced in JNK gain‐of‐function conditions (both Hep and *puc*) in heads of old, young or both ages, compared to controls. GO analysis was performed on FlyMine.org, using the indicated numbers of genes induced more that twofold with at least 10.0 FPKM. Note the higher functional diversity of genes induced by JNK in brains of young animals. (c) Selection of genes involved in glucose metabolism found to be induced in JNK gain‐of‐function conditions. Averages and *SEM* of FPKM values are shown. P‐values from Student's *t* test

The low steady‐state levels of G6P in *puc* mutants contrast with the observed faster rate of ^13^C‐G6P formation in these animals. To identify potential mechanisms of this effect of JNK signaling in the brain, we explored transcriptome changes downstream of JNK signaling using RNAseq analysis of brains from *puc* mutants and controls, as well as from animals over‐expressing the JNK Kinase Hemipterous (Hep) specifically in neurons (elav::Gal4, UAS::Hep) (Figure 4, Supporting Information Figure S5, Table S5). Among 217 genes induced by more than twofold in young flies with both genetic conditions compared to controls, we found a significant enrichment of genes encoding metabolic enzymes, including enzymes involved in glucose metabolism (Figure 4a–c, Supporting Information Figure S5). In old animals, in turn, elevated JNK activity boosted the expression of immune response genes, which are also induced in old wild‐type animals compared to their young counterparts (Figure 4a,b, Supporting Information Figure S5). Elevated JNK activity thus results in metabolic changes early in life and contributes to an elevated defense response later in life, consistent with previous findings (Libert et al., 2008).

**Figure 5:**
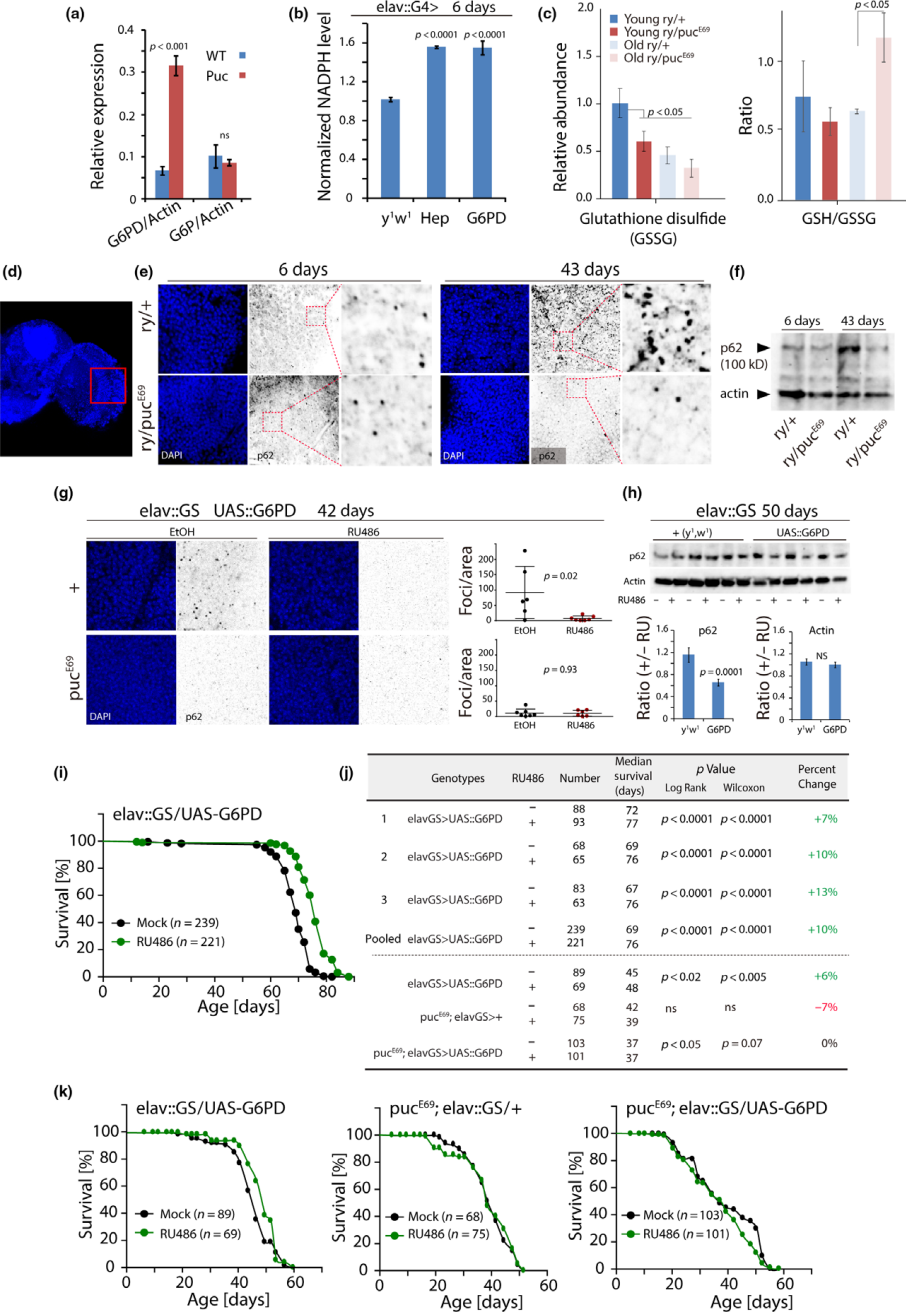
Increased G6PD expression promotes proteostasis and extends lifespan downstream of JNK signaling. (a) G6PD and G6P expression in the fly head measured by qRT–PCR. Expression values normalized to *actin 5C*. Averages and *SEM*, Student's *t*‐test. (b) NADPH concentrations in young Hep or G6PD over‐expressing heads, normalized to wild‐type controls. Averages and *SEM*, Student's *t*‐test. (c) Abundance of oxidized Glutathione (GSSG, measured by high‐resolution LC/MS) and GSH/GSSG ratio (measured enzymatically) in young and old *ry* and *puc* heterozygotes. (d) Adult brain with analyzed region outlined. DAPI staining was used to detect cell nuclei. (e) Representative images of young (6 days) and old (43 days) brains stained for p62 aggregates using immunohistochemistry. P62 foci increase in brains of old wild‐type (*ry*) flies, but not in brains of *puc^E69^* heterozygotes. (f) Western blot detecting p62 protein in brains of wild‐type (*ry*) and *puc^E69^* flies at different ages. (g) Reduced age‐related accumulation of p62 aggregates in wild‐type animals over‐expressing G6PD in neurons (elav::GS) for 42 days. Foci were quantified using ImageJ. Puc^E69^ heterozygotes show significantly lower levels of aggregates than wild‐type animals, and, accordingly, G6PD has no further effect in this background. (h) Western blotting of heads of animals over‐expressing G6PD for 50 days (using elav::GS). Averages and *SEM* of p62 and actin quantifications of three independent experiments shown in lower panels. ImageJ was used to measure protein band intensities, and ratios of siblings exposed or not to RU486 are shown as mean ± *SEM*. P‐values from Student's *t*‐test. (i) Survival curve for G6PD over‐expressing flies (elav::GS/UAS::G6PD). All females. Sizes (*n*) of sibling populations exposed to RU486 or vehicle (EtOH) are shown. (j) Summary of demographic parameters and lifespan statistics for (I and K). (k) Survival curves for G6PD over‐expressing flies in wild‐type (elav::GS/UAS::G6PD) or pucE69 heterozygous (pucE69/+; elav::GS/UAS::G6PD) background, as well as of pucE69 heterozygous controls (pucE69/+; elav::GS/+). All females. Sizes (n) of sibling populations exposed to RU486 or vehicle (EtOH) are shown. All flies were females

Using qRT–PCR, we confirmed that G6PD (glucose‐6‐phosphate dehydrogenase, encoded by the gene *Zwischenferment*, *Zw*), but not G6P (glucose‐6‐phosphatase), is highly induced in the brain of *puc* mutants (Figure 5a). The glucose‐6‐phosphatase complex hydrolyzes glucose‐6‐phosphate, generating free glucose, while G6PD oxidizes G6P by transferring hydride(s) to NADP+, forming NADPH, and resulting in carbon flux into the pentose phosphate pathway (PPP). The resulting increase in cytosolic NADPH is a critical defense against oxidative stress, reducing the concentration of oxidized glutathione (GSSG) (Bolanos & Almeida, 2010).

Supporting the notion that JNK and G6PD induce metabolic reprogramming of neurons by promoting carbon flux into the PPP, we found increased NADPH in heads of animals with increased JNK signaling activity, or increased expression of G6PD (Figure 5b). Similarly, we found reduced oxidized glutathione in *pucE69* heterozygotes and, especially in old *pucE69* heterozygotes, a significantly increased GSH/GSSG ratio (Figure 5c). At least part of the proteostatic effects of JNK activation may thus be mediated by G6PD induction, resulting in elevated carbon flux into the PPP, and consequent changes in NADPH and GSH levels. We tested whether this would contribute to proteostasis and measured the accumulation of the autophagy cargo receptor p62 and of cytosolic poly‐ubiquitinated protein aggregates (using the FK2 antibody) (Demontis & Perrimon, 2010) in the brain. p62 (also called ref(2)p) localizes to age‐induced protein aggregates, as well as to aggregates caused by reduced autophagic activity (Bjorkoy et al., 2009; Nezis et al., 2008). We observed a strong accumulation of p62 and of poly‐ubiquitinated aggregates in old wild‐type brains, and these phenotypes were significantly reduced in *puc^E69 ^*mutants (Figure 5d–f, Supporting Information Figure S6). These results further confirm that aging *puc^E69^*mutants maintain proteostasis in the brain more effectively than wild‐type flies. These phenotypes were recapitulated in animals in which G6PD was specifically over‐expressed in neurons (using an RU486‐inducible driver, elav::GS; Figure 5g,h), and this perturbation was also sufficient to extend lifespan (Figure 5i–k, Supporting Information Figure S6). This lifespan extension was not observed when G6PD was over‐expressed in *puc* mutant animals, indicating that the metabolic reprogramming seen in *puc* heterozygotes cannot further be optimized by G6PD over‐expression (Figure 5j,k).

## 3 DISCUSSION

**Figure 6:**
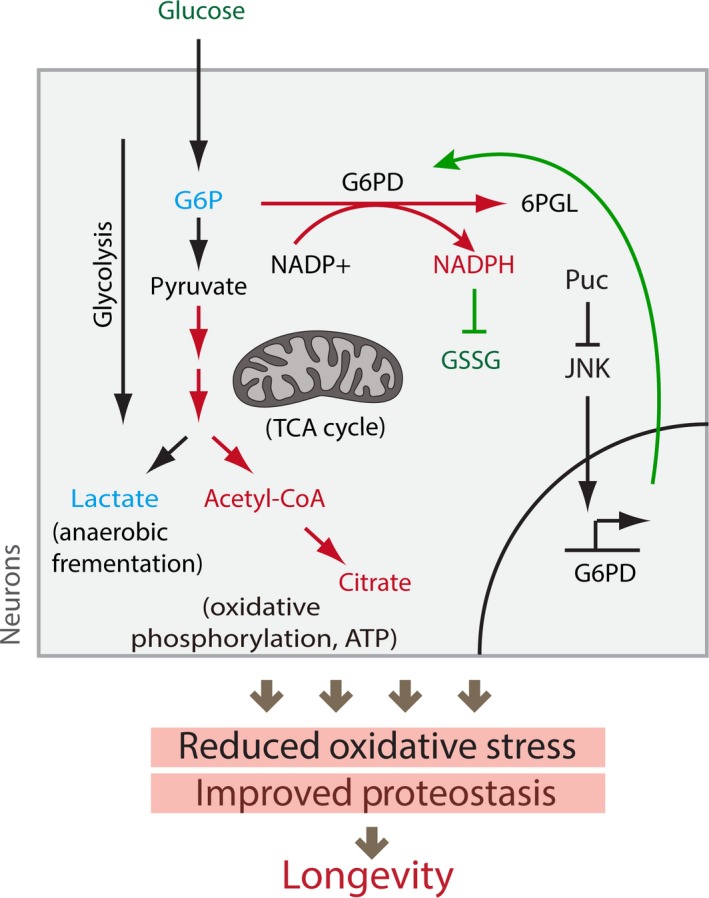
JNK‐mediated metabolic perturbation promotes proteostatic balance in the aging brain. Elevated JNK activity reduces IIS activity systemically, reducing glucose uptake, stimulating Foxo activity, and limiting the age‐related activation of the Tor pathway, thus reducing translation and maintaining higher levels of autophagy (Kapahi et al., 2010; Sarbassov, Ali, & Sabatini, 2005; Wang et al., 2005). At the same time, we find that JNK also induces the expression of G6PD in neurons, shifting metabolic carbon flux from glycolysis into the pentose phosphate pathway. The resulting increase in NADPH concentration in neurons increases the concentration of reduced glutathione, limiting protein damage, and associated protein aggregation. We propose that the coordinated control of IIS activity, Tor signaling, and metabolic flux by chronically elevated JNK activity thus results in broad proteostatic protection and lifespan extension

Our integration of large‐scale protein turnover analysis, metabolomic and metabolic flux analysis, as well as transcriptomic characterization and genetic perturbation, provides new insight into the link between metabolism, proteostasis, and longevity. Based on this insight, we propose a refined model of how activation of the JNK signaling pathway influences tissue health and longevity (Figure 6).

We have focused on the brain as a tissue in which metabolic changes in response to JNK activation occur in long‐lived postmitotic cells and in which these changes are thus likely to directly contribute to tissue health. The focus on JNK signaling allowed us to explore the cellular consequences of a perturbation that extends lifespan through modulation of stress‐responsive gene expression changes and that also directly influences metabolism by repressing the insulin/IGF signaling (IIS) pathway: JNK is activated by numerous stressors (including oxidative stress, ER stress, UV irradiation, DNA damage, or inflammatory cytokines) and can protect cells by inducing protective gene expression programs (Biteau et al., 2011; Karpac & Jasper, 2009; Karpac, Hull‐Thompson, Falleur, & Jasper, 2009; Oh et al., 2005; Wang et al., 2003, 2005 ). At the same time, JNK also extends lifespan by limiting IIS activity systemically, thus influencing sugar and lipid metabolism, cell growth and proliferation, protein translation, and autophagy (Biteau et al., 2011). Our results provide an integrated view of the cellular consequences of these diverse effects of JNK activation in the brain.

Critically, elevated JNK activity induces a change in neuronal carbon flux. This results in increased concentrations of NADPH and of reduced glutathione. Elevated NADPH is known to be a consequence of higher flux through the PPP and in turn increases the levels of reduced glutathione in neurons (Garcia‐Nogales, Almeida, & Bolanos, 2003; Legan et al., 2008). These changes are expected to result in reduced oxidative damage to proteins and, due to reduced accumulation of protein aggregates, in the higher protein turnover rates observed in JNK gain‐of‐function conditions. While JNK‐induced NADPH production thus likely directly prevents oxidation‐induced protein misfolding and aggregation (Dahl, Gray, & Jakob, 2015), we cannot rule out the possibility that changes in neuronal carbon flux also influence the activity of specific parts of the proteostasis network, including proteasome activity, autophagy flux, and/or mitochondrial proteostasis by, for example, influencing the activity of Lon protease (Lebeau, Rainbolt, & Wiseman, 2018). Interestingly, the master regulator of the antioxidant response, CncC (the *Drosophila* homologue of Nrf2), transcriptionally regulates proteasome levels in flies (Grimberg, Beskow, Lundin, Davis, & Young, 2011). It will therefore be of interest to explore the influence of CncC function on neuronal protein turnover and protein aggregate formation in the future.

Our isotope labeling study supports the notion that protein turnover rates decline in aging flies and provides a direct link between longevity and the maintenance of high protein turnover rates. This observation contrasts, however, with earlier work in which isotope labeling was used to compare somatic vs. germline investment in animals on dietary restriction (O'Brien, Min, Larsen, & Tatar, 2008). While this study did not directly assess protein turnover rates, the higher dietary nitrogen incorporation in fully fed vs. dietary restricted animals suggested a correlation of longevity with lower protein production or turnover. It will be of interest to explore the possibility that the metabolic changes elicited by dietary restriction impact longevity through mechanisms that are materially distinct from the mechanism of JNK‐induced longevity described here.

In *puc* mutants, carbon flux seems to be shifted through induction of G6PD, and we could phenocopy the effects of JNK gain‐of‐function conditions by increasing G6PD expression (which shifts carbon flux to the PPP). Transcriptional induction of G6PD is thus a central step in JNK‐induced proteostasis and lifespan extension. Supporting this notion, elevated G6PD expression has previously been associated with longevity in animals selected for long life (Luckinbill, Riha, Rhine, & Grudzien, 1990), and over‐expressing G6PD or reducing the expression of ribose‐5‐phosphate isomerase (an enzyme that catalyzes the isomerization of the downstream metabolite Ribulose‐5‐p to Ribose‐5‐p) in neurons have been found to be sufficient to extend fly lifespan, promote oxidative stress resistance, and limit polyglutamine toxicity (Legan et al., 2008; Wamelink, Struys, & Jakobs, 2008; Wang et al., 2012). It remains unclear whether elevated G6PD expression also recapitulates other aspects of JNK‐induced longevity, and it will be of interest to explore this. In particular, it will be of interest to assess whether elevation of neuronal G6PD, and neuronal metabolic reprogramming toward the PPP pathway, results in similar reduction in insulin signaling as JNK activation does. A limitation of our current study is the lack of *puc^E69^*/G6PD genetic epistasis experiments for longevity, as we were unable to generate healthy animals in which *puc^E69^*/+ was combined with brain‐specific G6PD‐RNAi or loss‐of‐function alleles. A concerted effort to generate G6PD loss‐of‐function tools should be able to resolve this and allow addressing the question of whether G6PD is essential for all JNK‐mediated longevity and healthspan effects, or whether it is only responsible for aspects of these phenotypes.

The connection between glucose metabolism, oxidative stress, and aging has also emerged in recent years in work using *C. elegans*, in which it was shown that glucose and glycogen metabolism influences longevity through the production of NADPH and reduced glutathione (Gusarov et al., 2017). Importantly, this study highlights the deleterious effects of too much glucose‐mediated cellular NADPH production, as it promotes the reduction in glutathione to a point where cellular stress responses are impeded. It is interesting in this context that in *Drosophila*, it was recently shown that longevity of *dilp2* mutants is dependent on the activity of glycogen phosphorylase, the enzyme that promotes glucose release from glycogen stores (Post et al., 2018).

Glucose is central to energy metabolism in the brain, both in astrocytes and neurons, and defects in glucose metabolic pathways have been documented in neurodegenerative disorders (Mathur, Lopez‐Rodas, Casanova, & Marti, 2014): Pyruvate levels are increased in patients with multiple sclerosis (McArdle, Mackenzie, & Webster, 1960), while impaired GAPDH and ECT components were found in both AD and HD patients (Brooks et al., 2007; Chandrasekaran et al., 1994; Mazzola & Sirover, 2001; Regenold, Phatak, Makley, Stone, & Kling, 2008). How metabolic deregulation is involved in functional changes in the aging brain, however, is largely unknown. Recent studies using an HD fly model found that increasing glial energy metabolism by over‐expressing uncoupling proteins, or modulating neuronal metabolism by over‐expression of Glut1 or G6PD in neurons can alleviate the pathology of flies transgenic for a pathogenic huntingtin protein (HD93Q) (Besson, Alegria, Garrido‐Gerter, Barros, & Lievens, 2015; Besson, Dupont, Fridell, & Lievens, 2010). The effects of G6PD over‐expression in this model are consistent with our findings.

While providing insight into fundamental mechanisms influencing aging and proteostasis, our findings are thus also expected to further inform studies exploring the relationship between metabolic deregulation in neurons, changes in neuronal proteostasis, and age‐related neurodegeneration. As used here, comprehensive approaches to characterize protein turnover rates, steady‐state metabolite concentrations, and metabolic flux rates, applied to long‐lived animal models, are thus likely to help develop detailed models for the biochemical adjustments common in and required for lifespan‐extending perturbations.

## 4 MATERIALS AND METHODS

### 4.1 Chemicals and standards

Glucose‐6‐phosphate, citric acid, lactate, and ammonium acetate were obtained from Sigma‐Aldrich (St. Louis, MO, USA). For metabolomics studies, HPLC‐grade solvents acetonitrile and methanol were purchased from Fisher Scientific (Pittsburgh, PA, USA) and VWR (Radnor, PA, USA). Deionized water was generated in‐house for mobile phase preparation. For proteomics studies, HPLC solvents including acetonitrile and water were obtained from Burdick & Jackson (Muskegon, MI, USA). Reagents for protein chemistry including iodoacetamide, dithiothreitol (DTT), ammonium bicarbonate, and formic acid were purchased from Sigma‐Aldrich. Proteomics grade trypsin was from Promega (Madison, WI, USA).

### 4.2 Flystocks and maintenance

*w^1118^, y'w’,* OreR, *ry^506^*, elav::GeneSwitch, and elav::Gal4^c155^ were obtained from the Bloomington *Drosophila* Stock Center. UAS::G6PD was generously provided by Dr. William C. Orr. Puc^E69^ was a gift from Dr. Dirk Bohmann.

Female flies were used throughout. Flies were reared on yeast/molasses‐based food (agar [13.8 g], molasses [22 g], malt extract [75 g], dry yeast [Inactivated, 18 g], corn flour [80 g], soy flour [10 g], propionic acid [6.2 ml], methyl 4‐hydroxybenzoate [2 g], EtOH [7.2 ml], H_2_O [1 L]) at 25°C, and 65% humidity on a 12‐hr light/dark cycle.

### 4.3 Isotopic labeling and sample preparation for proteomics by LC/MS

#### 4.3.1 Yeast labeling

About 5 ml of minimal media (0.5% ^15^N‐ or ^14^N‐labeled ammonium sulfate [(NH_4_)_2_SO_4_], 2% sucrose, 0.17% yeast nitrogen base without amino acids, and ammonium sulfate [Difco, # 233520]) was used to grow yeast from a single colony overnight at 30°C. One millilitre pre‐labeled yeast in ^15^N or ^14^N medium was then inoculated into 1 L minimal yeast media in a 2.5‐L flask overnight at 30°C. Yeast was pelleted at 5,800 *g* for 10 min and used either immediately to make fly food, or stored at −20°C for a few months after PBS washes.

#### 4.3.2 Fly labeling

Young (5 days) and old (35 days) flies were fed on standard sugar/yeast food before being switched on ^15^N‐ or ^14^N‐labeled yeast fly food (1.5% agar, 10% sugar, 10% ^15^N or ^14^N pre‐labeled yeast, 0.3% propionic acid, and Tegosept). Food was changed every 2–3 days during the 8 days of labeling time.

#### 4.3.3 Sample preparation for LC/MS

Fly heads were collected by freezing flies with liquid nitrogen in a 15‐ml tube after 8 days of labeling. Tissue samples were homogenized in RIPA buffer by motorized pestle and centrifuged down for 10 min at 16,000 *g* to get rid of cell debris. Subsequently, ~100 µg of total protein lysate from each sample was combined with 4× LDS Gel Sample Loading Buffer (Invitrogen), and samples were reduced with 500 mM DTT at 60°C for 1 hr, and alkylated 250 mM iodoacetamide at RT in the dark for 1 hr. Then samples were loaded on a 1D SDS‐PAGE gel, separated, and stained with Biosafe Coomassie G‐250. Each gel lane was divided into 10 pieces according to the molecular weight (10–250 kDa), and bands were excised, see cut‐out scheme (Supporting Information Figure S2). All gel pieces were then proteolytically digested with trypsin to yield tryptic peptides for LC‐MS/MS analysis.

### 4.4 Proteomic mass spectrometric analysis

Samples were analyzed by reverse‐phase HPLC‐ESI‐MS/MS using an Eksigent Ultra Plus nano‐LC 2D HPLC system (Dublin, CA) connected to a quadrupole time‐of‐flight TripleTOF 5600 mass spectrometer (SCIEX). Typically, mass resolution for MS1 scans and corresponding precursor ions was ~35,000, while resolution for MS2 scans and resulting fragment ions was ~15,000 (“high sensitivity” product ion scan mode). Data acquisition was performed in data‐dependent mode (DDA) on the TripleTOF 5600 to obtain MS/MS spectra for the 30 most abundant precursor ions following each survey MS1 scan. Specifically, samples were acquired by reverse‐phase HPLC‐ESI‐MS/MS using an Eksigent Ultra Plus nano‐LC 2D HPLC system (Dublin, CA) which was directly connected to a quadrupole time‐of‐flight (QqTOF) TripleTOF 5600 mass spectrometer (SCIEX, Concord, CAN). Briefly, after injection, peptide mixtures were transferred onto the analytical C18‐nanocapillary HPLC column (C18 Acclaim PepMap100, 75 µm I.D. × 15 cm, 3 µm particle size, 100 Å pore size, Dionex, Sunnyvale, CA, USA) and eluted at a flow rate of 300 nl/min using the following gradient: at 5% solvent B in A (from 0 to 13 min), 5%–35% solvent B in A (from 13 to 58 min), 35%–80% solvent B in A (from 58 to 63 min), at 80% solvent B in A (from 63 to 66 min), with a total runtime of 90 min including mobile phase equilibration. Solvents were prepared as follows, mobile phase A: 2% acetonitrile/98% of 0.1% formic acid (v/v) in water, and mobile phase B: 98% acetonitrile/2% of 0.1% formic acid (v/v) in water.

### 4.5 Data accession

The mass spectrometric raw data and spectral libraries associated with this manuscript may be downloaded from MassiVE at ftp://massive.ucsd.edu/MSV000080118 (MassIVE ID: MSV000080118).

### 4.6 Protein Identifications and proteome‐wide determination of ^15^N incorporation ratios and clearance rates

MS/MS peak lists were extracted using the program MSConvert (Kessner, Chambers, Burke, Agus, & Mallick, 2008) from the LC‐MS/MS raw data files corresponding to the unlabeled flies. Peptide identification was conducted by Protein Prospector (version 5.10.12) against the *Drosophila melanogaster* Uniprot database. To this database, a randomized version was concatenated to allow determination of false discovery rates. The search parameters were the following: species = *Drosophila melanogaster*; enzyme specificity = trypsin; allowed missed cleavages = 1; fixed modification = carbamidomethylation; variable modifications = acetylation on protein N terminus, glutamine on peptide N‐terminal to glutamic acid, methionine loss from protein N terminus, methionine loss from protein N terminus and acetylation, methionine oxidation; maximal number of variable modifications = 2; precursor mass tolerance = 50 ppm; fragment mass tolerance = 0.6 Da; peptide expectation cutoff = 0.05. The match of sequences from the decoy database (normal + random) indicated that the false discovery rates (FDR) for this expectation cutoff value were <1% for all analyses. A complete list of protein identifications and supporting statistics are provided in Figure 2 and Supporting Information Table S1.

Using the Protein Prospector database search results, the following information was gathered for each peptide ion with an expectation value <0.05: monoisotopic mass to charge ratio (*m/z*), charge (*z*), retention time (RT), assigned protein, and sequence. The data were tabulated as a text file. Using the program MSConvert, MS1 spectra were extracted from all raw data files obtained from the labeling time‐course. The MS1 spectra were centroided, and the resulting peaklists were used for further analysis. Using a script written in Java (*mssplice*, Zhang et al., 2014), the following set of analyses was sequentially conducted for each tabulated peptide obtained from the database search:

1. Numbers of nitrogen atoms were determined for each peptide sequence.
2. The possible set of isotopic *m/z* values for each peptide was determined, assuming a range of 0.0–1.0 ^15^N incorporation ratios.
3. Using a series of overlapping 30‐s RT windows, ranging from two minutes prior to two minutes passed the experimentally determined RT, MS1 spectra ranging from the monoisotopic *m/z* to the maximum possible isotopic *m/z* (assuming 100% ^15^N incorporation) were collected and averaged. The total intensity of all possible peptide isotopic *m/z* values was summed and plotted as a function of RT to generate a chromatogram for each peptide. The chromatogram was fitted with a Gaussian function to determine the peak width. MS1 spectra within the determined RT peak width and the *m/z* window were summed, and the aggregated spectra were used for further analysis.
4. The extracted MS1 peptide spectra were fitted with a probabilistic combinatorial isotopic distribution model that considers the spectra as the sum of two isotopic populations (labeled and unlabeled). The ^15^N enrichment of the unlabeled population was set as a constant to its natural abundance. The ^15^N enrichment of the labeled population and the ratio of labeled and unlabeled populations were considered as the two fitted variables. Least‐square fitting to the model was used to obtain the following information: the fraction ^15^N incorporation of the labeled population, the ratio of labeled and unlabeled populations, goodness of fits (coefficient of determination—*R* ^2^) to predicted isotopomer distributions for labeled and unlabeled populations. Only spectra that had *R* ^2^ values of greater than 0.8 for both populations were used for further analysis.
5. For each peptide ion where more than four unique time‐points passed the above criteria, the fraction labeled values were fitted to a single exponential equation to determine the clearance rate.
6. Peptide level labeled population measurements were aggregated for each homologous group of proteins. Peptides with sequences that were shared among multiple homologous groups were not considered.
7. The aggregated data were fitted with a single exponential equation to determine protein clearance rates. A complete list of measured turnover rates is provided in the Figure 2 and Supporting Information Table S2

### 4.7 Statistical significance testing

All mass spectrometric data were searched as described above using Protein Prospector against a *Drosophila melanogaster* Uniprot database in combination with a decoy database. For the database searches, an FDR cutoff of less than 1% was applied for all protein identifications. The statistical significance (*p* value) of global differences in turnover rates between different ages and genetic backgrounds was established by Mann–Whitney *U* tests as described in the figure legends.

### 4.8 Ontology/pathway analysis

A list of gene ontology (GO) (Gene Ontology Consortium, 2015) terms associated with the analyzed proteins was obtained from https://geneontology.org (March 2015). For the localization plot shown in Figure 2e, the proteomic data mapped to the GO terms GO:0005634 (nucleus), GO:0005829 (cytosol), GO:0005739 (mitochondria), and GO:0005615 (extracellular) were collected and the distribution of the measured turnover rates was analyzed. Additional Ontology Analysis was performed using the web‐based program DAVID v.6.7 (The Database for Annotation, Visualization, and Integrated Discovery), which was used for functional analysis and protein ontology analysis (Huang da et al., 2008).

### 4.9 LC/MS for steady‐state metabolites

#### 4.9.1 Fly metabolite extraction

Metabolite extracts were prepared from 10 flies by adding 50 µl of 50% methanol standard followed by 1‐min sonication on ice; 75 µl of chloroform was then added to the sample and mixed for 1 min. The samples were centrifuged at 10,000 *g* for 20 min at 4°C to separate the organic and aqueous layers. The aqueous layer was removed and transferred into 150‐µl vial inserts for LC‐MS/MS analysis.

#### 4.9.2 HPLC‐MS (Metabolomics)

For high‐resolution (accurate‐mass) HPLC‐MS analysis, HPLC was performed using an Agilent 1260 Infinity LC system fitted with following modules: u‐degasser (G1322A), binary pump (G1312B), thermoregulated column compartment (G1330B), and HiPALS autosampler (G1367E). Chromatographic separation of fly extracts was performed on a Phenomenex Luna NH_2_ (2.0 mm × 150 mm, 3.0 µM) column. The solvent system consisted of A = 20 mM ammonium acetate pH 9.5 with 5% acetonitrile and B = acetonitrile. The starting gradient conditions were 95% B at a flow rate of 0.3 ml/min. The following gradient program was used: 0–20 min, 95%–10% B, 25–30 min 10% B, and 30.1–35 min 95% B. Samples were kept at +4°C, and the injection volume was 10 µl.

High‐resolution MS1 was performed using an Agilent 6520 QTOF mass spectrometer fitted with a Dual‐Spray Electrospray Source (ESI). The instrument was operated at a mass resolution of ~20,000 for TOF MS1 scan using 2GHx extended dynamic range mode. The ionization parameters were set as follows: gas temperature (TEM) 350°C; drying gas, 9 L/min; Vcap, 2,500 V; nebulizer gas, 35 psi; fragmentor, 125 V; and skimmer, 65 V. MS1 acquisition was operated in the negative ion scanning mode for a mass range of 50–1,000 *m/z*.

The HPLC‐MS data were acquired using Agilent MassHunter Workstation (B.05.00). Agilent MassHunter Qualitative Analysis Software (B.07.00), Mass Profiler Professional (B.12.0), and Microsoft excel 2007 (Redmond, WA, USA) were used for an in‐depth MS1 analysis of fly extracts. To perform a comparative quantitation of metabolites, peak area were assigned using Agilent MassHunter Qualitative Analysis Software in combination with the Find by Formula (FBF) algorithm. Peak areas were normalized by total protein.

### 4.10 Statistical analysis

All data were presented as means ± *SEM*. Comparisons between groups were performed using 2‐tailed Student's *t* test.

### 4.11 Metabolic flux analysis by ^13^C‐glucose injection (sample preparation)

A 65 µl of 0.125 g/ml ^13^C‐Glucose (Cambridge Isotope Lab, CLM‐1396) was directly injected into the hemolymph using the Nanoject II Auto‐Nanoliter Injector (Drummond Scientific Company). Blue *dye *(FD&C *Blue *#1) was coinjected with ^13^C‐Glucose to monitor the injection. Fly heads were then collected at different time‐points (5, 15, 30, 60, and 120 min) by being flash frozen in liquid nitrogen. Total amount of protein measured by BCA was used to normalize each sample.

### 4.12 HPLC‐MS ^13^C‐metabolic flux analysis

HPLC was performed using a Shimadzu UFLC prominence system fitted with following modules: CBM‐20 A (Communication bus module), DGU‐A_3_(degasser), two LC‐20AD (liquid chromatography, binary pump), SIL‐20AC HT (autosampler), and connected to a Phenomenex Luna NH_2_ (2.0 mm × 150 mm, 3.0 µM) column. The solvent system was A = 20 mM ammonium acetate pH 9.5 with 5% acetonitrile and B = acetonitrile. The starting gradient conditions were 95% B at a flow rate of 0.3 ml/min. The following gradient program was used: 0 to 20 min, 95%–10% B, 25–30 min 10% B, and 30.1–35 min 95% B. Samples were kept at +4°C, and the injection volume was 10 µl.

Mass spectrometric analysis for ^13^C flux experiments was conducted using negative ion electrospray ionization in the multiple reaction monitoring mode (MRM) on a SCIEX 4000 QTRAP (Redwood City, CA, USA) mass spectrometer fitted with a TurboV^TM^ ion source. The ionization parameters were set as follows: curtain gas (CAD); 20 psi; collision gas: medium; ion spray voltage (IS): −4,500 V; Temperature (TEM): 550°C; Ion source Gas 1 (GS1); 60 psi; and Ion source Gas 2 (GS2): 50 psi. The compound parameters were established using the appropriate standards. The compound parameters were set as follows: entrance potential (EP): −8.0 V; and collision cell exit potential (CXP): −5 V. Analyst^®^v1.6.1 (SCIEX) was used for all data acquisition, method development, and in‐depth analysis of the HPLC‐MS data, specifically for calculating the peak areas for the identified features from fly extracts. Peak areas were normalized by total protein (Table 1).

**Table 1:** Compound parameters and MRM transitions for the following glycolytic and mitochondrial metabolites

Metabolite	Q1 (*m/z*)	Q3 (*m/z*)	Declustering potential (DP)	Collision energy (CE)	RT
^12^C glucose 6‐P	259.0	97.0	−53.0	−18.0	20.8
^13^C glucose 6‐P	265.0	97.0	−53.0	−18.0	20.8
^12^C citrate	191.0	111.0	−53.0	−18.0	24.4
^13^C citrate	197.0	116.0	−53.0	−18.0	24.4
^12^C lactate	89.0	43.0	−38.0	−17.0	12.7
^13^C lactate	92.0	45.0	−38.0	−17.0	12.7

### 4.13 Western blot

Heads from female flies were collected by freezing flies in liquid nitrogen and mechanically separating heads from bodies. Heads were homogenized in RIPA buffer with protease inhibitors for 30 min and then were centrifuged 12,000 rpm for 20 min for the supernatant collection. The total protein was measured (Pierce BCA protein assay kit, Prod # 23227) to estimate the loading amount before protein sample buffer added. Primary antibodies (rabbit anti‐p62, 1:2000, Pircs et al., 2012; rabbit anti‐b‐actin, 1:3,000, Cell Signaling #4967) were applied, followed by HRP‐conjugated anti‐rabbit and chemiluminescence, according to manufacturer instructions.

### 4.14 NADPH measurement

Fly heads of each genotype were collected by being frozen in liquid nitrogen. Thirty frozen fly heads were washed with cold PBS and homogenized with 150 µl NADP/NADPH extraction buffer provided by NADP/NADPH quantification kit (BioVision, Cat.# K347‐100). NADPH concentration of each sample was measured according to the protocol in NADP/NADPH quantitation kit (BioVision, Cat. # K347‐100) and was normalized by its own total protein (Pierce BCA protein assay kit, Prod # 23227). At least three independent samples were analyzed for NADPH measurement.

### 4.15 Immunostaining and microscopy

Fly brain was dissected in PBST (0.1% Triton in PBS), fixed for 20 min at room temperature on nutator in 4% formaldehyde in PBST, washed three times with PBST for 30 min at room temperature on nutator, blocked with blocking buffer (5% NGS in 0.1% PBT) for 30 min, and incubated with primary antibodies in blocking buffer (rabbit anti‐p62, 1:200, Pircs et al., 2012, mouse anti‐polyubiquitin, FK2, Enzolifescience, 1:200) for two night at 4°C on nutator, followed by second antibodies for 2 hr at room temperature.

Fluorescent secondary antibodies were purchased from Jackson ImmunoResearch Laboratories. DNA was stained using DAPI. Confocal imaging was performed on a Zeiss LSM700 confocal microscope and processed using ImageJ and Adobe Illustrator.

### 4.16 RNAseq analysis

Total RNA of young (5 days) or old (40 days) fly heads was isolated using *RNA* Miniprep *Kit* (Zymo Research, R1054). Total RNA was used as template to generate RNAseq libraries for Illumina MiSeq sequencing. Reads were mapped to the *Drosophila* genome (release 5.2) using a standard Tuxedo suite pipeline, and RPKM (reads per kbp per million reads) values were recorded for each gene. Differential expression was determined in Excel, and Gene Ontology was determined using Flymine.org.

### 4.17 Lifespan analysis

Thirty virgins (elav::GS) were crossed to 15–20 UAS‐G6PD (generously provided by Dr. William C. Orr) males. Female progeny of these crosses according to genotype was sorted into cages (70–90 flies/cage at 3–4 days after the first fly hatched and then aged at 25°C and 65% humidity on a 12‐hr light/dark cycle). About 100 μl of 5 mg/ml solution of RU486 or vehicle (80% ethanol) was added on the top of a food vial and dried overnight before fed to flies. Food was changed every other day. Demographic data were analyzed using Prism statistical software.

For the lifespan of *puc^E69^* flies, *puc^E69^*, *ry^506^*/+, and OreR flies were crossed to *ry^506^/ry^506^* on standard sugar/yeast food (1.8% yeast). Female progeny of these crosses were sorted into cages (70–90 flies/cage) and reared on fly food containing high yeast (3.2% yeast), low yeast (0.8%), or still on moderate yeast (1.8%). Food was changed every 2–3 days. Statistical significance was analyzed using Prism software.

### 4.18 GSH/GSSG assay

Measurements of glutathione and glutathione disulfide were made using the GSH/GSSG Ratio Detection Assay Kit from Abcam (ab138881), according to manufacturer's instructions. Briefly, 20 whole female heads per sample were flash frozen at 6 days (young) or 42 days (old). Samples were resuspended in 150 μl PBS with 0.5% Triton X‐100 and dissociated with a motorized pestle. Debris was removed by centrifugation and deproteinated with trichloroacetic acid (25 μl/sample) and sodium bicarbonate (80 μl 7% w/v). Samples were diluted 4× for the GSH assay, and 16× for GSSG. All samples were kept on ice using the enzymatic reaction step. The reaction mixture was incubated 45 min, then read with a BioTek fluorescence plate reader.

## CONFLICT OF INTERESTS

The authors declare no competing interests.
