# Supplementary material for: JNK modifies neuronal metabolism to promote proteostasis and longevity
Source: Aging Cell. 2019 Feb 27;18(3):e12849. doi: 10.1111/acel.12849 (PMC6516429; doi:10.1111/acel.12849)
Supplement: Supplementary file 1 [file ACEL-18-e12849-s001.docx]

**Wang et al., Supplemental Information**


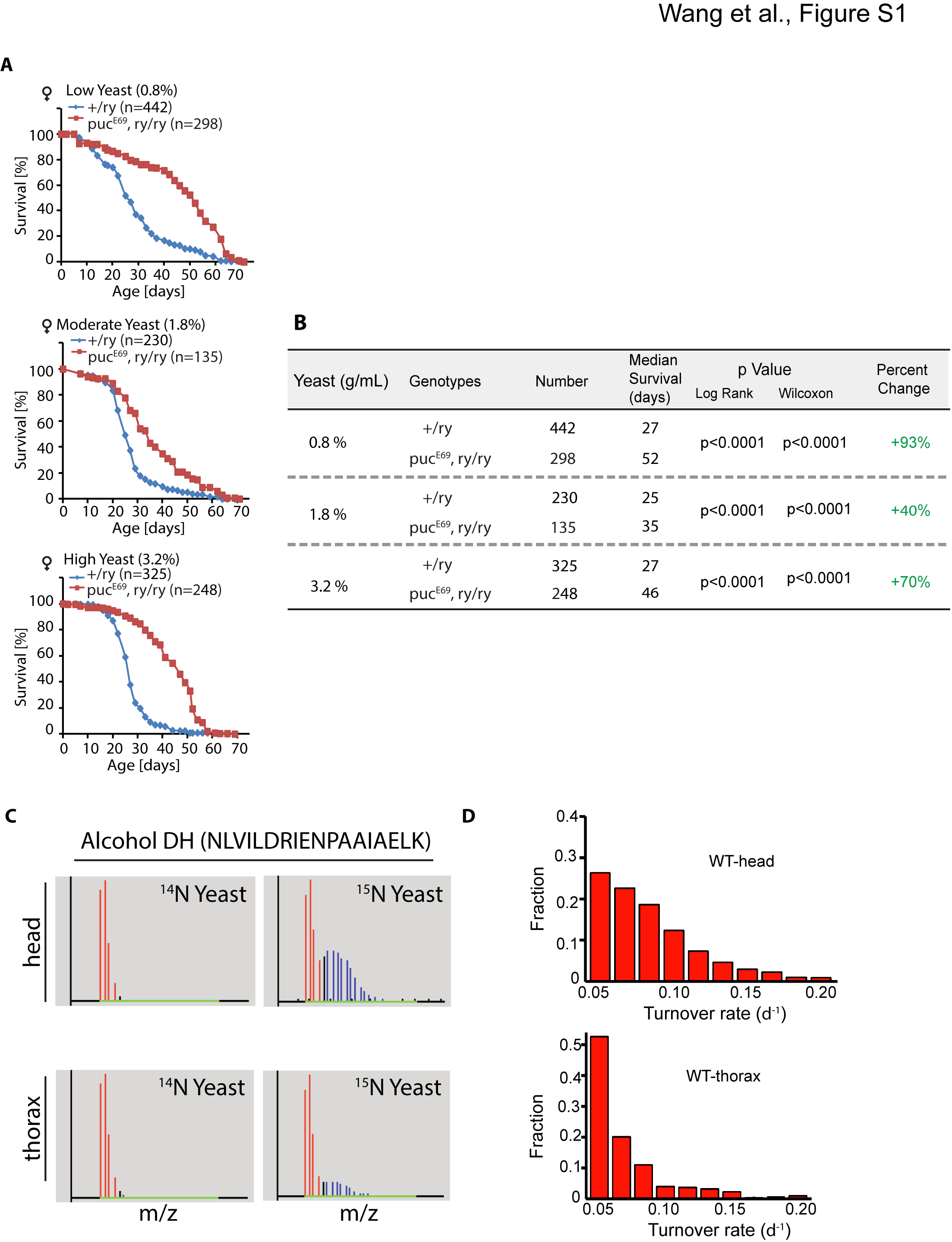


**Figure S1: PucE69 flies with increased JNK activity can extend lifespan on different dietary conditions**

1. Reducing one copy of *puc* (*puc^E69^, ry/ry*) results in robust lifespan extension compared to isogenic wild-type controls (ry/+). Lifespan extension is independent of dietary conditions. Diets used contained varying concentrations of yeast (Low yeast: 0.8%, moderate yeast: 1.8%, high yeast: 3.2%) with constant concentrations of sugar (22 g/L molasses).
2. Table summarizing parameters and lifespan statistics of the flies in Fig S1A

**
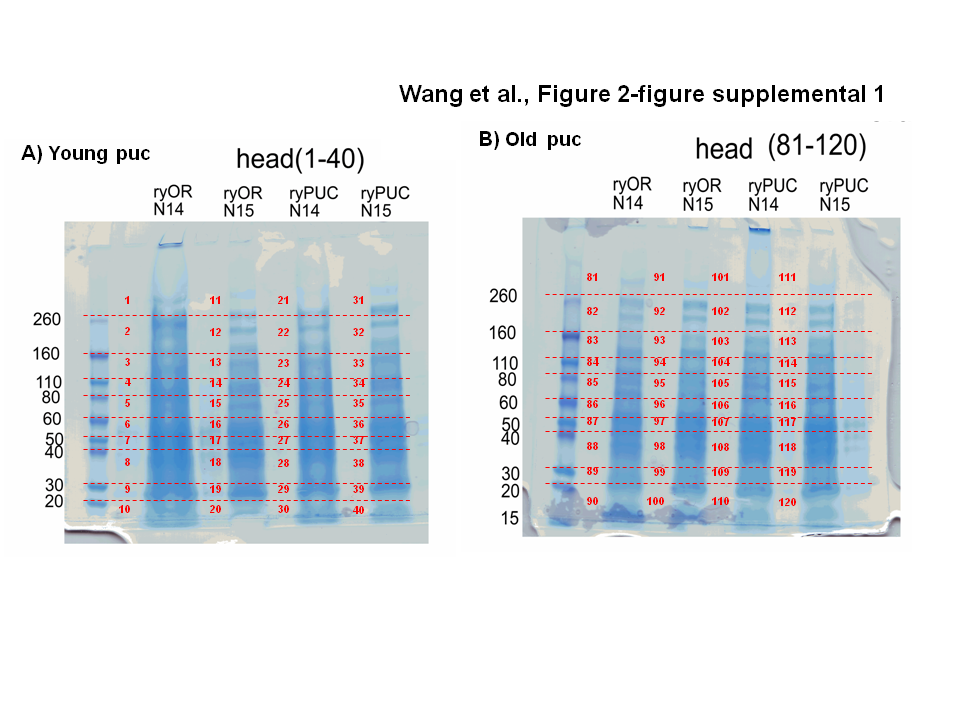
**

**Figure S2: Global analysis of protein turnover rate in fly head**

1D SDS-PAGE gel cutout scheme indicating the bands that were cut out for in gel digestion and subsequent LC-MS/MS analysis. Gel band numbering as indicated in the cutout scheme was correlated with the raw file naming.


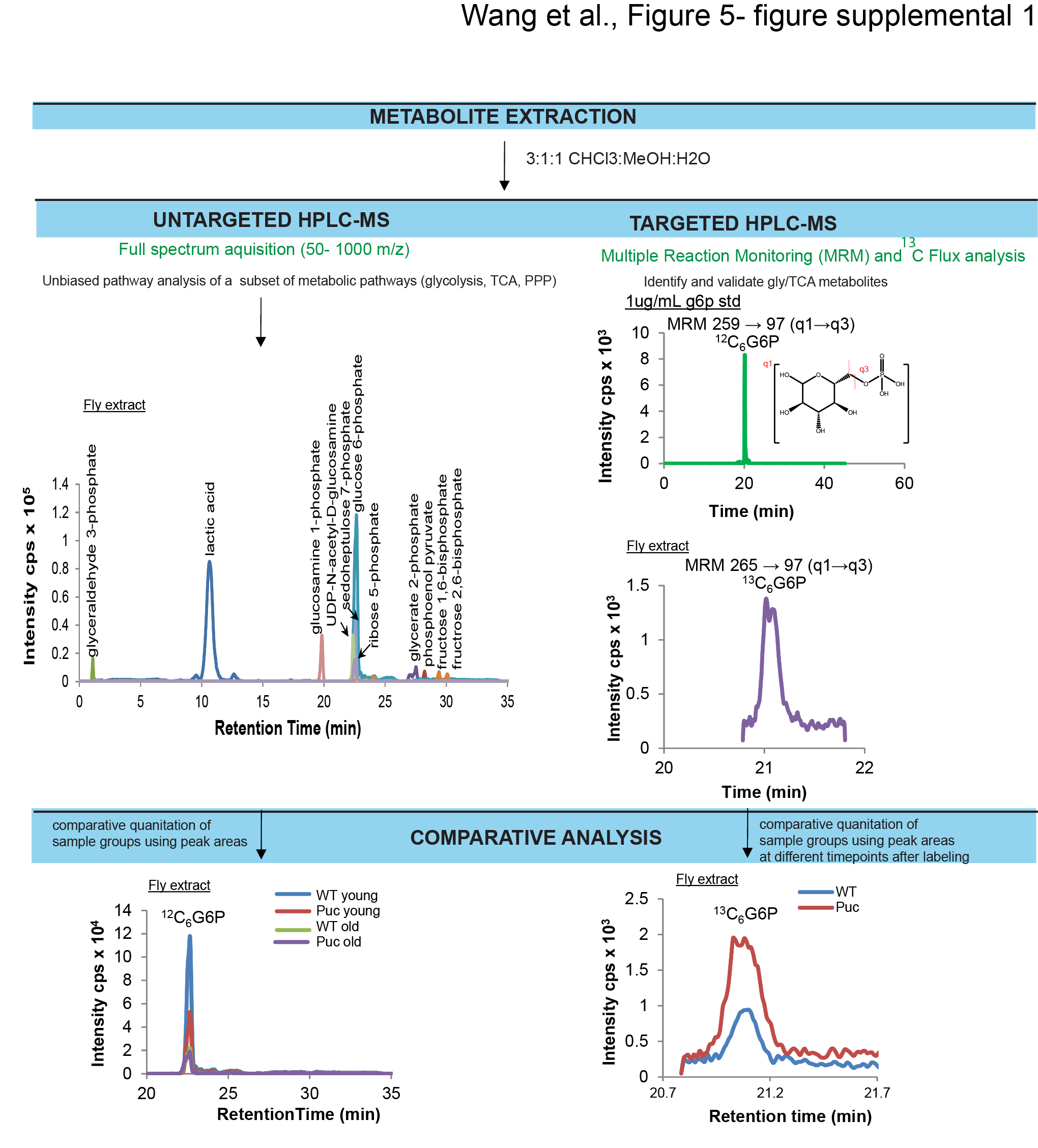


**Figure S3: Outline of experimental strategy for metabolites identified and analyzed by HPLC-MS**

Metabolite extraction was isolated by three-phase methanol–water–chloroform partitioning before being loaded on HPLC-MS instrument. A peak integration method was applied to quantitatively measure untargeted (for metabolite profiling) or targeted (for the downstream effectors of ^13^C metabolic flux) metabolomics. See also the detailed description in the section of Materials and Methods.


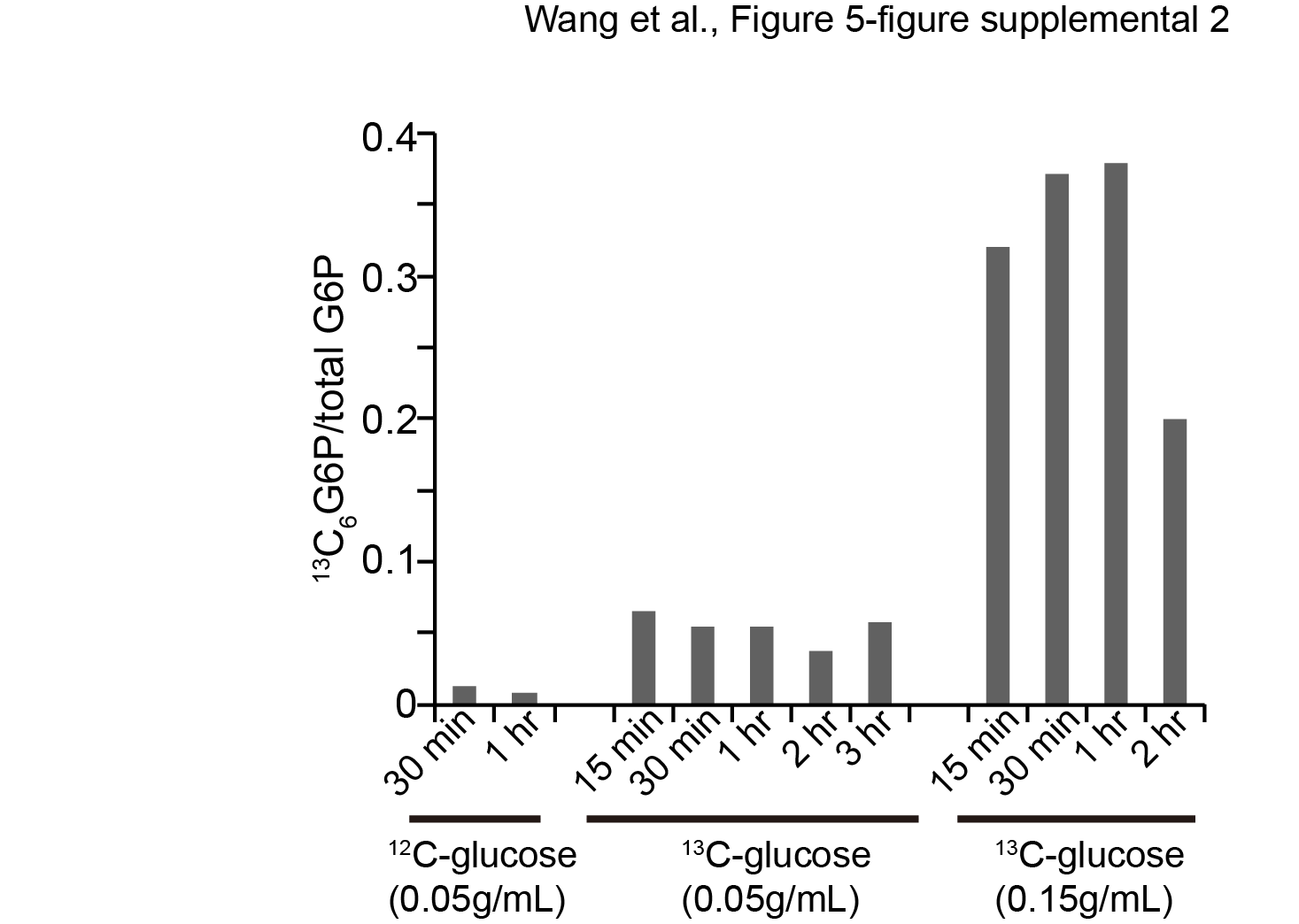


**Figure S4: ^13^C labeled Glucose-6-phosphate detection in fly head**

The same volume (69 µL) of ^12^C-glucose (control, 0.05 g/mL), ^13^C glucose (lower concentration, 0.05 g/mL) or ^13^C glucose (higher concentration, 0.15 g/mL) was injected into hemolymph. Fly head was then collected at indicated time points to detect the circulating metabolite ^13^CG6P traced by ^13^C.


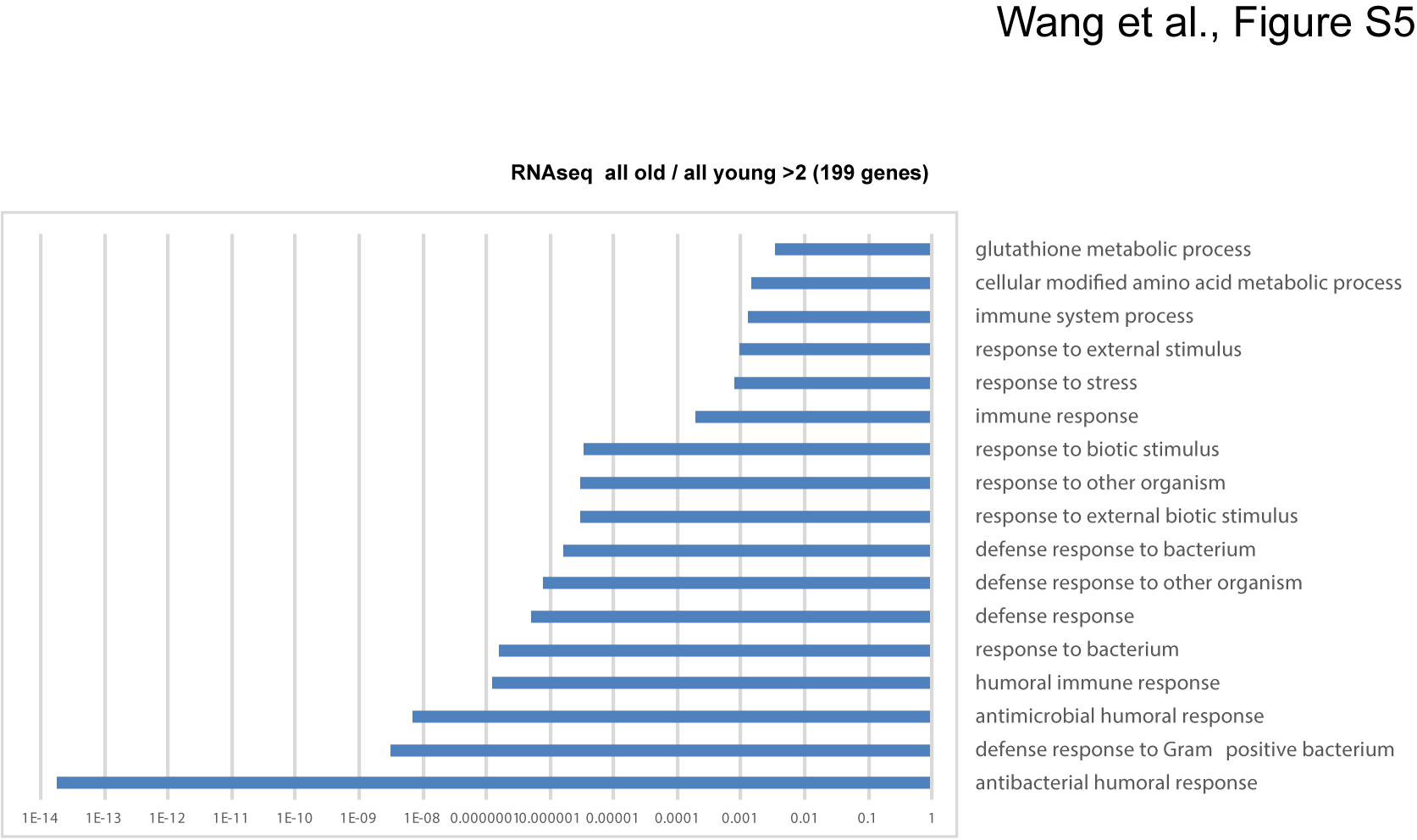


**Figure S5: GO analysis of genes induced in aging wild-type heads**

A list of 199 genes identified by RNAseq analysis was selected for GO analysis in the conditions of (1) gene was induced more than 2 fold in old wild-type fly heads compared to the young ones, and (2) FPKM value of genes was at least 10.0


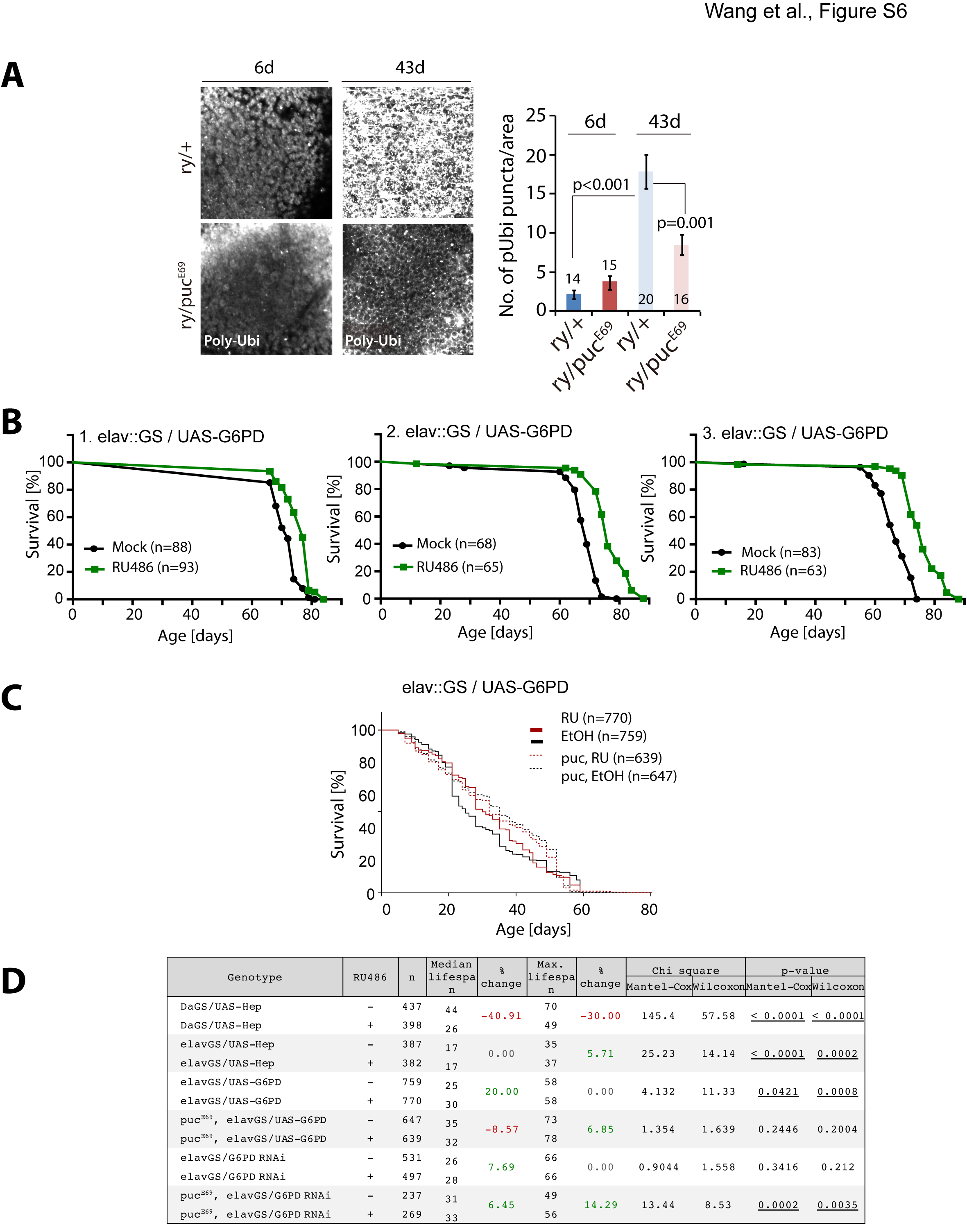


**Figure S6: G6PD overexpression in the brain extends lifespan**

Lifespan curves of G6PD over-expressing flies (elav::GS/UAS::G6PD) from each individual experiment. Pooled lifespan curved of over-expressing flies was shown in Figure 6I. The lifespan statistics was summarized in Figure 6J.
